# Adherence to Vitamin D Supplementation during Infancy—A Single Pediatric Primary Practice Retrospective Study

**DOI:** 10.3390/pediatric15040059

**Published:** 2023-11-02

**Authors:** Jerko Vucak, Jeronim Matijevic, Ivan Pivac, Josko Markic

**Affiliations:** 1 Primary Health Care Pediatric Practice, Sibenik 22000, Croatia; vucakj@gmail.com; 2 Department of Pediatrics, University Hospital of Split, Split 21000, Croatia; jeronimmatijevic@gmail.com; 3 School of Medicine, University of Split, Split 21000, Croatia; ivan.pivac4@gmail.com

**Keywords:** vitamin D, supplementation, adherence, infant

## Abstract

The risk of vitamin D deficiency is high in infants. Therefore, potential vitamin D deficiency should be prophylactically treated with vitamin D supplementation. Achieving good adherence to recommended prophylactic regimens is the goal of every primary pediatrician. The aim of this paper was to establish whether Croatian infants receive recommended prophylactic doses of vitamin D regularly. We analyzed the prescription rate of vitamin D preparation during the first year of life in one pediatric primary practice. Our research has shown, for the first time in Croatia, that there is low treatment adherence. Only 7.6% of infants received the recommended doses of vitamin D. The percentage of infants in the moderately irregular adherence group was 19.3%. There was no statistical difference regarding urban or rural place of living or parents’ educational level. Based on these findings, a comprehensive public health campaign is needed to improve adherence to vitamin D supplementation during infancy. Also, further studies on larger samples and on a national level are warranted.

## 1. Introduction

Two of the most important forms of vitamin D (VD) are vitamin D2 (ergocalciferol) and vitamin D3 (cholecalciferol). Cholecalciferol is formed in the epidermal layer of the skin under the influence of UV rays from its precursors. Both forms of vitamin D can be found in various dietary products. Vitamin D from the diet or from skin synthesis is biologically inactive. In the liver, it undergoes the process of 25-hydroxylation, and in the kidneys, by the action of the enzyme 1-alpha hydroxylase, it becomes biologically active as 1,25(OH)_2_ vitamin D. Because of its steroid form and way of acting in cells, it is sometimes considered a hormone [1,2]. VD is crucial in the homeostasis of calcium and phosphate and, consequently, in bone metabolism. In the pediatric population, vitamin D deficiency can cause rickets by decreasing the mineralization of developing bones and reducing bone stiffness [3]. Suboptimal mineralization of existing (old) bone during the remodeling process causes osteomalacia and occurs in the bones of adults or adolescents with closed growth plates [2]. The global incidence of rickets and osteomalacia is rising, partly due to failing prevention policies and lack of effective implementation strategies [2]. Hypocalcemia can cause seizures, tetany, and dilated cardiomyopathy, while hypophosphatemia can lead to hypotonia and delayed development. Beside this, VD has many other important roles, including the regulation of the immune system. It also has an impact on cerebrovascular diseases and even autism spectrum disorders [4,5,6,7]. It was also found that hypovitaminosis D is very common in children with multisystem inflammatory syndrome (MIS-C), which may develop after COVID-19 and influences the severity of the disease. Patients with lower serum VD concentrations have been found to have worse systolic and diastolic function of the left ventricle [8].

The most important means of VD synthesis is UV exposure [9]. In addition to time spent outdoors, the synthesis depends on various other factors, like skin pigmentation, degree of latitude, season, the amount of cloud cover, percentage of exposed skin, sunscreen usage, etc. In the normal infant diet, there is an insufficient amount of VD, especially in exclusively breastfed infants, due to the low content of vitamin D in breastmilk [10,11]. The amount of VD available in infant formula milk is insufficient as well. Also, low maternal vitamin D reserves are passed on to the fetus. Therefore, the reason why VD supplementation in the infant population is introduced on a global level and endorsed by various medical societies is to ensure adequate levels of VD and to protect the neonate/infant from developing complications from this form of deprivation.

Nutritional rickets and osteomalacia are fully preventable via universal supplementation in infants. Most developed countries have VD supplementation policies in place. However, some countries are less successful in their implementation. Due to limited natural dietary sources of VD; limited sun exposure, especially during the first months of infancy, which may increase the risk of skin cancer; and the fact that adequate sunshine exposure for the cutaneous synthesis of VD is not easily determined, the American Academy of Pediatrics revised the recommendations to ensure adequate VD status to include all infants, older children, and adolescents. Since 2008, it has been recommended that all of them have a minimum daily intake of 400 IU of vitamin D beginning soon after birth [11]. They also recommended that, in children with increased risk of VD deficiency, such as those with chronic fat malabsorption and those chronically taking antiseizure medications, higher doses may be necessary to achieve normal VD status, and the status should be determined with laboratory tests [11]. The European Academy of Pediatrics recommends dietary VD intake for children and adolescents without risk factors of 400 IU/day during the first year of life, and 600 IU/day after the first year of life, up to the age of 18 years. They also recommend higher VD intakes for infants, children, and adolescents with risk factors for VD deficiency. In accordance with the European Food Safety Authority, an upper safety limit for VD supplementation was set at 1000 IU/day for infants, 2000 IU/day for children ages 1 to 10 years, and 4000 IU/day for children and adolescents ages 11 to 17 years [4,12]. The Committee on Nutrition of the French Society of Pediatrics, however, recommends higher doses of VD supplementation for infants (breast-fed infants: 1000–1200 IU/day; children younger than 18 months of age receiving milk supplemented with vitamin D: 600–800 IU/day; children younger than 18 months of age receiving milk not supplemented with vitamin D: 1000–1200 IU/day) [13].

In Croatia, parents are advised to start peroral VD supplementation for their infant with VD doses of 400–1000 IU/day during the first year of life. The treatment is fully covered by government health insurance through the prescription given to them by the primary pediatrician. Although there are numerous vitamin D preparations available, the vast majority of patients’ parents receive a prescription for Plivit^®^ D3 (Pliva Hrvatska d.o.o., Zagreb, Croatia). In addition to this, exclusively breastfed children are advised to receive a combination of vitamin D and vitamin K during the first 3 months of life (BabytolD3+K1^®^, 4U Pharma, Chrzanow, Poland). After that period, for the rest of their infancy, they continue to receive a VD preparation (Plivit^®^ D3 or similar).

The rising trend of preventable diseases in the twenty-first century, including rickets, should be reversed. Existing recommendations provide guidance for both health care professionals and policymakers. All of them should strive to make VD supplements available to all children within their community. But the goal can be achieved only if the parents also follow supplementation recommendations. Several epidemiological studies have shown a high prevalence of VD deficiency among infants regardless of age, ethnicity, and geographical location, ranging between 2.7% and 45% [14,15,16]. Therefore, it is evident that universal supplementation is significantly associated with good adherence to supplements according to the primary pediatrician’s recommendation. The aim of this study was to analyze the adherence to VD supplementation during infancy in a single pediatric practice in Croatia.

## 2. Materials and Methods

This research included all children born in the period from 1 January 2019 until 31 December 2019 who were patients in a single pediatric primary practice and for whom vitamin D supplementation with Plivit^®^ D3 was prescribed. Patients receiving another form of VD supplementation (*n* = 12) were excluded. In all of them, Oleovit D3 (Fresenius Kabi Austria GmbH, Linz, Austria) was prescribed based on their personal request. There were no other exclusion criteria.

Patients were divided into two groups. Group 1 included infants for whom, at the time of the first pediatric examination (at the age of 1 month), Plivit^®^ D3 was prescribed, with recommendation to the parents to administer it orally in a daily dose of 800 IU (4 drops) until the age of one year. A prescription was issued for one bottle, and the parents were instructed to contact their primary pediatrician when the following bottle was needed. At all subsequent regular pediatric check-ups (2, 3, 4, 6, and 9 months of age), the parents were informed of the need for regular administration of VD therapy. All of them indicated that they would administer or were already administering the therapy as prescribed. At the age of 1 year, the patient’s medical charts were reviewed to determine how many bottles of VD were used in that period. Medical charts indicate not only that the medication was prescribed but also that it was collected from the pharmacy. Based on the amount of VD in the bottle (10 mL/200 drops) and assuming it was given regularly at 4 drops/day, 1 bottle of VD was expected to last for 50 days. Each patient’s therapy began after the first regular pediatric check-up at the age of 1 month (30 days). Since there are 335 days until the age of 1 year, if taken regularly and according to prescription, it was calculated that each patient needed 6.7 bottles of VD. VD adherence was considered regular if 6 or 7 bottles were used in the observed period, moderately irregular if 5 bottles were used, and irregular if ≤4 bottles were used.

Group 2 included infants who received BabytolD3+K1^®^ during the first 3 months of life and then continued to receive Plivit^®^ D3 for the following 9 months. All parents indicated, at regular pediatric check-ups (3, 4, 6, and 9 months of age), that they would administer or were already administering the therapy as prescribed. If taken regularly and according to prescription, it was calculated that each patient needed 5.5 bottles of VD. VD adherence was considered regular if 5 or 6 bottles were used in the observed period, moderately irregular if 4 bottles were used, and irregular if ≤3 bottles were used.

The patients were additionally divided into two groups based on their place of living (urban, rural). Patients living in the towns of Šibenik, Vodice, and Skradin were designated to the urban group, while others were designated to the rural group. To investigate the impact of parents’ education on adherence to VD administration, the patients were divided into three groups based on their adherence to VD supplementation and the level of education of the parents: VSS—university education; SSS—high school education; and NSS—those with elementary-school-level education. Since all children lived with both parents and there was no “head of the household”, mothers’ and fathers’ levels of education were analyzed for each infant.

Statistical analyses were conducted using Microsoft Office Excel version 16.0 (Microsoft Corporation, Redmond, WA, USA) and IBM SPSS Statistics version 23.0 (IBM Corp., Armonk, NY, USA). Categorical variables were depicted as absolute counts accompanied by percentages, while continuous variables were presented as means along with their corresponding standard deviations (M ± SD). The analysis of categorical variables was conducted using the Chi-square test, while disparities in continuous variables were evaluated using the independent samples *t*-test. A *p*-value < 0.05 was considered statistically significant.

## 3. Results

A total of 145 infants fulfilled the inclusion criteria, with 76 (52.4%) being males. Out of them, 104 (58 males) were designated for Group 1, and 41 (18 males) were designated for Group 2. During the study period, 467 bottles of Plivit^®^ D3 were consumed. On average, 3.2 ± 1.5 bottles were prescribed per infant: 3.1 ± 1.6 for boys and 3.3 ± 1.4 bottles for girls (*p* = 0.517). Overall, only 11 (7.6%) infants had regular adherence to a VD supplementation and received the recommended dose of VD supplementation: 8.7% in Group 1 and 4.9% in Group 2, *p* = 0.109. The percentage of infants in the moderately irregular adherence group was in total 19.3%: 23.1% in Group 1 and 9.8% in Group 2. (Table 1). A noteworthy observation is that, when considering both regular and moderate users, only a quarter of the infants (26.9%) adhered to the vitamin D supplementation regimen.

Of the total number of children, 94 (64.8%) lived in urban areas and 51 (35.2%) lived in rural areas. Regarding adherence to vitamin D supplementation during infancy, there was no significant difference among the groups based on place of living (*p* = 0.932) (Table 2).

When the parents’ (*n* = 290) educational levels were analyzed, 85 of them were designated to the VSS group (29.3%), 199 to the SSS group (68.6%), and 6 to the NSS group (2.1%). Regarding adherence to vitamin D supplementation during infancy, there was no significant difference among the groups based on educational level of the parents (*p* = 0.606) (Table 3).

## 4. Discussion

Being aware of the importance of vitamin D supplementation in the infant population, the aim of this study was to investigate adherence to VD supplementation during infancy in a single primary pediatric practice in Croatia. VD supplementation is considered safe and effective for improving VD status. Prevention policies are based on reliable data. Many countries, including ours, are promoting an infant VD supplementation policy. However, a paucity of national data across the whole world and a total lack of any data for Croatia regarding adherence to VD supplementation creates a challenge in designing appropriate improvements to existing policies. We found that only 7.6% of infants had regular adherence to VD supplementation and received the recommended dose of VD supplementation. We assumed that a certain number of doses of the Plivit^®^ D3 would be skipped for various reasons (forgetfulness, illness, travel, etc.). Therefore, we added the regular users to the moderately irregular ones and found that, for only 26.9% infants, it can be assumed that they received sufficient VD supplementation doses, with significantly more irregular users. The results were below our expectations and led to the question of whether it even made sense to continue implementing this national preventive program in the same way.

A study performed by Uday et al. distributed a questionnaire on country-specific VD supplementation policy and child health care programs, socioeconomic factors, policy implementation strategies, and adherence among the members of the European Society for Pediatric Endocrinology, Bone and Growth Plate Working Group, and other specialists. Good (≥80% of infants), moderate (50–79%), and low adherence (<50%) to supplements were reported by 59% (17/29), 31% (9/29), and 10% (3/29) of the countries, respectively. The lowest adherence, ranging between 5% and 20%, was reported in the United Kingdom, while the highest rate (98%) was reported in Austria and Hungary [17]. The authors concluded that national supplementation policies are in place across Europe, but their implementation has been neglected. Also, their results showed that relatively simple implementation strategies are associated with good adherence. Specifically, integrating the monitoring of adherence into existing prevention programs such as child health surveillance visits (‘red book’, passports) increases adherence. Croatia wasn’t a part of this research, making the results of our study even more valuable. A study conducted in 2020 by Simon et al. found that 27.1% of United States of America (USA) infants in 2009–2016 followed the vitamin D supplementation guidelines, while non-breastfeeding infants were more likely to meet guidelines than breastfeeding infants. They also concluded that vitamin D adherence positively correlated with socio-economic status and with a college graduate head of household [18]. More precisely, they found that breastfeeding infants in families with a head of household who was a college graduate were more likely than those in families with a head of household with a lower level of education to meet guidelines (26.2% vs. 10.7%; *p* < 0.05) [18]. Simon et al. used a standardized interview to obtain information on vitamin D intake, while we calculated the exact amount of vitamin D supplementation by checking the number of prescribed bottles collected from the pharmacy. The results of our study did not find a correlation with the educational levels of the parents (*p* = 0.606) and no correlation between place of living (*p* = 0.932) and adequate adherence to vitamin D supplementation during infancy. A previous study from the USA showed that only 4% to 7% of the infants were receiving oral VD supplementation. The prevalence of oral supplement use ranged from 5% to 13% in the breastfed group, while only 1% to 4% of the infants in the formula-fed group were receiving an oral supplement. The authors estimated that, among breastfed infants, only 9% to 13% were receiving enough vitamin D to meet the 2008 AAP recommendation [19]. Higher rates were found in Canada. A cohort study of infants born at a hospital in Montreal in 2007–2008 found that 74% of breastfeeding infants met VD intake guidelines [20]. Reasons for these low and concerning rates are not fully known. One possible reason could be that physicians are not as concerned about rickets as they were before [18]. Other reasons may include a physician’s failure to successfully present the need for VD supplementation to the parents or failure to adhere to physician recommendations by parents or caregivers.

To reverse these current negative trends, it is important to initiate a public health campaign with the aim of improving adherence to VD supplementation during infancy. A national campaign on the importance of vitamin D supplementation should be similar to national campaigns that already took place in Croatia, like the ones about the importance of breastfeeding or the campaign “First three years are the most important”. Hemmingway et al. published an analysis of adherence to the national infant VD supplementation policy in Ireland, which was introduced in 2010. They showed that almost all participants were supplemented with VD at some point during infancy, and there was a high initiation rate of supplementation at birth, indicating widespread awareness of the Irish VD supplementation policy. They also found substantial increases in supplement use since the policy’s introduction, suggesting that the policy has become well established [21].

In addition, medical professionals (pediatricians, pediatric nurses, etc.) should make more effort to educate parents about the importance of vitamin D supplementation during infancy. Pediatricians need additional education as well [22]. Recently, we conducted a study aimed at evaluating the knowledge and practice of European pediatricians concerning VD and hypovitaminosis D and their adherence to relevant guidelines [22]. ESPGHAN’s and the national guidelines were the most frequently used. Participants predominantly answered the questions regarding the definition of hypovitaminosis D and VD supplementation correctly, especially in infants. The reason for this is that pediatricians, especially general pediatricians, have more experience with VD supplementation in infants, as they usually prescribe supplemental VD to all infants. However, in older children, they prescribe it only for those with a clinical indication or low sun exposure. Therefore, regarding older children, their knowledge of supplemental VD therapy decreased. They also showed the least knowledge regarding the cut-off point for the initiation of the therapy and therapeutic doses used to treat confirmed hypovitaminosis D [22]. As a solution, we think that uniform guidelines should be created, and those guidelines should be promoted by national or local pediatric associations. Additionally, further education about VD, hypovitaminosis D, and VD therapy standardization should be encouraged by European, national, and local pediatric associations [22]. If all additional procedures and better implementation of existing policies prove to be insufficient, there is also the possibility of giving bolus doses of vitamin D [23]. Children might receive 50,000 IU of vitamin D in a bolus dose every two months under regular pediatric control. Parents would be able to choose between the old approach and this new approach to supplementing vitamin D. Also, a recent review suggested that exclusive high-dose VD supplementation of 6400 IU/day or >100,000 IU on a monthly schedule provided to mothers and a proper administration given to the child can both be deemed suitable approaches for the prevention of VD deficiency in exclusively breastfed infants [24].

This study has some limitations. It was conducted on a small number of participants and included a single pediatric primary practice. Therefore, results cannot be considered representative of infants from Croatia and should be checked on a larger and more representative sample. Also, information on infants’ feeding was not collected, nor were the VD levels in the blood of infants, to see whether there was a significant clinical impact of the irregular adherence to VD supplementation on VD levels. In a Cochrane Review conducted in 2020 by Tan et al., which analyzed 19 studies, it was found that supplementation of vitamin D in infants increases 25-OH vitamin D levels in the blood but it is unclear if it affects bone mineral content [25]. Finally, although all the parents indicated that they were administering the VD according to the prescription, there is always a level of uncertainty regarding the actual administration of the preparation to the child.

## 5. Conclusions

This is the first analysis of adherence to the national infant vitamin D supplementation policy in Croatia. We found that only 7.6% of infants met the vitamin D intake guidelines. Place of living (urban or rural) or parents’ educational level had no impact on adherence to VD supplementation. We demonstrated the need for prompt public health initiatives and additional effort by health care professionals, but also more political effort, in the implementation of efficient VD supplementation programs to prevent symptomatic VD deficiency and protect the most vulnerable members of our society. Also, having in mind the limitations of this research, further studies are warranted.

## Figures and Tables

**Table 1:** Number of used Plivit^®^ D3 bottles during infancy (*n* = 145).

Vitamin D Bottles Used	Group 1	Group 2
	(*n* = 104)	(*n* = 41)
6 or 7	9 (8.7%)	0
5	24 (23.1%)	2 (4.9%)
4	16 (15.4%)	4 (9.8%)
3	24 (23.1%)	19 (46.3%)
2	16 (15.4%)	11 (26.8%)
1	15 (14.4%)	5 (12.2%)

Group 1—infants who were taking Plivit^®^ D3 from the age of 1 month; Group 2—infants who were taking Plivit^®^ D3 from the age of 3 months.

**Table 2:** Impact of place of living on adherence to vitamin D supplementation during infancy (*n* = 145).

Adherence to Vitamin D Supplementation	*n*	Urban Area (*n* = 94)	Rural Area (*n* = 51)	*p*-Value
Regular users	11 (7.6%)	7 (7.4%)	4 (7.8%)	0.932 *
Moderately irregular users	28 (19.3%)	19 (20.2%)	9 (17.6%)	
Irregular users	106 (73.1%)	68 (72.3%)	38 (74.5%)	

* Chi-square test.

**Table 3:** Impact of parent’s educational level on adherence to vitamin D supplementation during infancy (*n* = 290).

Adherence to Vitamin D Supplementation	N	Parental University Education (VSS) (*n* = 85)	Parental High School Education (SSS) (*n* = 199)	Parental Elementary School Education (NSS) (*n* = 6)	*p* Value
Regular users	22 (7.6%)	7 (8.2%)	15 (7.5%)	0 (0.0%)	0.606 *
Moderately irregular users	56 (19.3%)	12 (14.1%)	43 (21.6%)	1 (16.7%)	
Irregular users	212 (73.1%)	66 (77.6%)	141 (70.9%)	5 (83.3%)	

* Chi-square test.

## Data Availability

Research-anonymized data is available upon reasonable request from the first author.
